# Preliminary Characterization of a Leptin Receptor Knockout Rat Created by CRISPR/Cas9 System

**DOI:** 10.1038/srep15942

**Published:** 2015-11-05

**Authors:** Dan Bao, Yuanwu Ma, Xu Zhang, Feifei Guan, Wei Chen, Kai Gao, Chuan Qin, Lianfeng Zhang

**Affiliations:** 1 Key Laboratory of Human Disease Comparative Medicine, Ministry of Health, Institute of Laboratory Animal Science, Chinese Academy of Medical Sciences & Comparative Medical Center, Peking Union Medical College, China.; 2 Key Laboratory of Human Disease Animal Model, State Administration of Traditional Chinese Medicine, Institute of Laboratory Animal Science, Chinese Academy of Medical Sciences & Comparative Medical Center, Peking Union Medical College, China.

## Abstract

Leptin receptor, which is encoded by the diabetes (*db*) gene and is highly expressed in the choroid plexus, regulatesenergy homeostasis, the balance between food intake and energy expenditure, fertility and bone mass. Here, using CRISPR/Cas9 technology, we created the leptin receptor knockout rat. Homozygous leptin receptor null rats are characterized by obesity, hyperphagia, hyperglycemia, glucose intolerance, hyperinsulinemia and dyslipidemia. Due to long-term poor glycemic control, the leptin receptor knockout rats also develop some diabetic complications such as pancreatic, hepatic and renal lesions. In addition, the leptin receptor knockout rats show a significant decrease in bone volume and bone mineral density of the femur compared with their wild-type littermates. Our model has rescued some deficiency of the existing rodent models, such as the transient hyperglycemia of *db*/*db* mice in the C57BL/6J genetic background and the delayed onset of glucose intolerance in the Zucker rats, and it is proven to be a useful animal model for biomedical and pharmacological research on obesity and diabetes.

Leptin receptor (*Lepr*), which is encoded by the diabetes (*db*) gene and is highly expressed in the choroid plexus, regulates energy homeostasis, the balance between food intake and energy expenditure, fertility and bone mass, by binding to leptin, which is encoded by the obese (*ob*) gene^1^,^2^,^3^,^4^. Mapping of the gene’s chromosomal locations in rodents revealed that mutations in *Lepr* were the basis for obesity/diabetes in rodents and humans^4^.

Accordingly, *Lepr*-deficientmice, the autosomal recessive diabetes mutants (*db*/*db*), have severe early-onset obesity, extreme insulin resistance and develop diabetes^5^,^6^,^7^, which have been used in studies on obesity and diabetes. The rat equivalent of the *db*/*db* mouse is the Zucker rat, also known as the *fa*/*fa* rat^8^, which carries a spontaneous autosomal mutation in the *Lepr* gene and develops a similar phenotype of hyperphagia leading to morbid obesity^9^,^10^, glucose intolerance and insulin resistance^5^,^11^,^12^. Although the Zucker rats have often been used to study type 2 diabetes-like syndromes, they do not develop the full phenotype of type 2 diabetes. For example, the Zucker rats do not present with a typical high blood glucose level^10^.

The Clustered Regularly Interspaced Short Palindromic Repeats (CRISPR)/CRISPR-associated (Cas) system is a RNA-based adaptive immune system in bacteria and archaea and now has been engineered as RNA-guided endonucleases for genome editing. A type II CRISPR system functions by the CRISPR RNA (crRNA) interacting with a *trans*-activating crRNA (tracrRNA) to form a crRNA-tracrRNA duplex which could be replaced with a single guide RNA (gRNA), then leading to the formation of Cas9 protein-containing ribonucleoprotein complexes that recognized and cleave a target DNA sequence^13^,^14^. Unlike other engineered nucleases, such as zinc finger nucleases and transcription activator-like effector nucleases, the CRISPR/Cas9 system does not require the engineering of specific protein pairs for each target site, which has made the CRISPR/Cas9 system develop into convenient genome editing tool for producing gene knockout models of many species^15^,^16^,^17^,^18^,^19^. Here, we generated the *Lepr* knockout rats using the CRISPR/Cas9 system. The *Lepr* knockout rats were obese, sterile, and diabetic and had decreased bone mineral density, which would expand the suite of animal models for biomedical and pharmacological research on obesity and type 2 diabetes.

## Results

### Generation of leptin receptor knockout rat using CRISPR/Cas9 system

**Figure 1:**
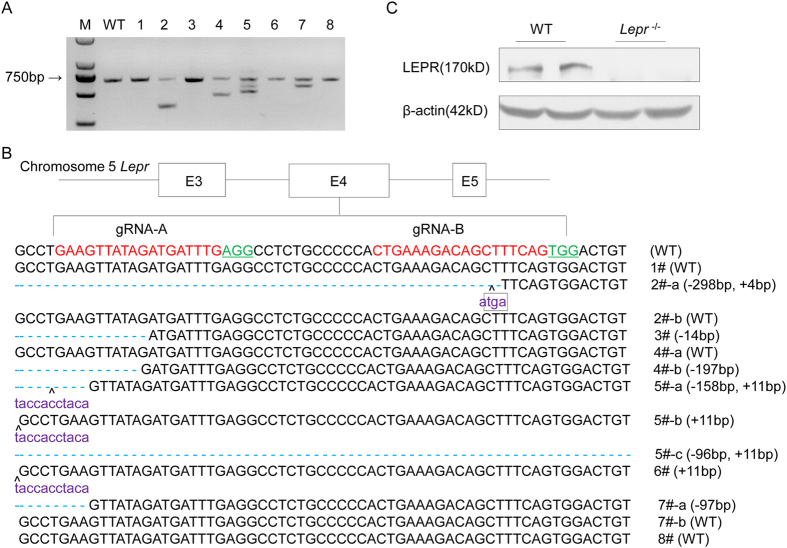
Generation of Leptin receptor knockout rat using CRISPR/Cas9 system. (**A**) Target loci of *Lepr* were amplified using genomic DNA templates from founders. M: DNA molecular weight marker DL2000; WT: Template DNA was replaced with wild-type genomic DNA; 1-8: Founder rats generated by microinjection. (**B**) PCR products of the targeted fragment in the *Lepr* in rats were sequenced. The protospacer adjacent motif (PAM) sequence was underlined and highlighted in green; the targeting sites were red; the insertions were purple, lower case; insertions (+) or deletions (−) were shown to the right of each allele. The E3, E4 and E5 represents exon 3, exon 4 and exon 5 of *Lepr* respectively. (**C**) Protein level of LEPR in the liver tissues of WT littermates and *Lepr*^−/−^ rats were detected by western blot, using β-actin as normalization.

Two gRNAs targeting *Lepr* exon 4 were transcribed *in vitro*. A mixture of Cas9 mRNA (20 ng/ul) and gRNA (10 ng/ul per gRNA) were microinjected into the cytoplasm of zygotes of Sprague Dawley (SD) rats. A total of 60 injected zygotes were transferred to 2 pseudopregnant female SD rats, and 8 pups were born. The PCR amplification of the targeting loci showed that four rats (founder 2, 4, 5 and 7) had *Lepr* deletion (Fig. 1A). Further sequencing confirmed that six rats (founder 2–7) had a frame shift mutation (Fig. 1B). Founder 2 was chosen to establish a colony (designated as *Lepr*^−/−^), which carried a 298-bp deletion from No. 90043 bp to 90341 bp in the *Lepr* genome DNA sequence (NC_005104.4) and a 4-bp insertion, and resulted in a termination codon TGA, deleting 997- amino acid of LEPR. Western blot analysis of total protein from liver tissue of the *Lepr*^−/−^ rats confirmed the absence of LEPR (Fig. 1C).

### Leptin receptor knockout induced obesity and hyperphagia

**Figure 2:**
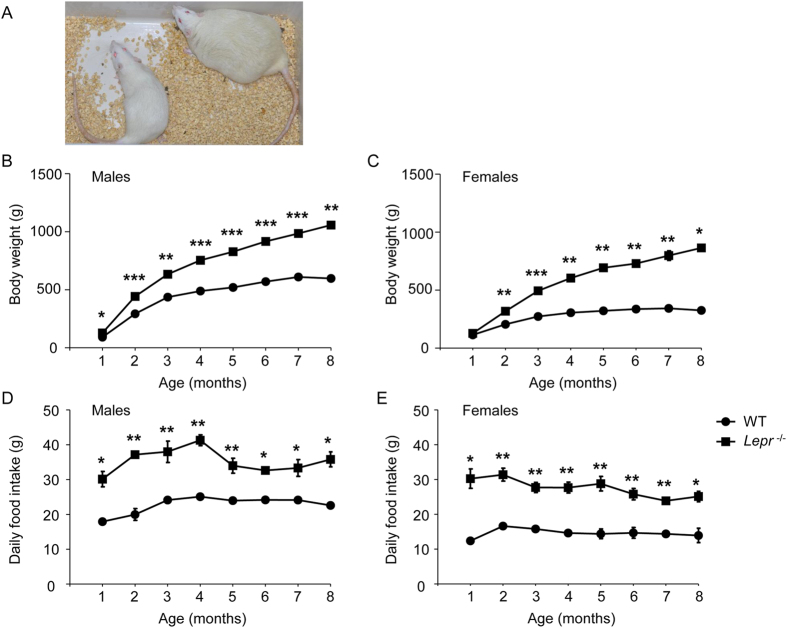
Body weight and daily food intake measurement of Leptin receptor knockout rat. (**A**) A picture of a WT littermate and a *Lepr*^−/−^ rat at 8 months of age. (**B**,**C**) Body weight was measured over 8 month for male WT littermate (n = 19), male *Lepr*^−/−^ rat (n = 12), female WT littermate (n = 13) and female *Lepr*^−/−^ rat (n = 15). (**D**,**E**) Daily food intake was measured over 8 month for male WT littermate (n = 12), male *Lepr*^−/−^ rat (n = 8), female WT littermate (n = 8) and female *Lepr*^−/−^ rat (n = 8).*P < 0.05, **P < 0.01, ***P < 0.001 *versus* WT littermate rats.

The body weight was measured from 1 to 8 months of age. The male *Lepr*^−/−^ rats emerged with severe early-onset obesity as early as 1 month of age and were approximately 60% heavier at 8 months of age compared with their wild-type (WT) littermates (Fig. 2A,B, n = 12, *P* = 0.002). The female *Lepr*^−/−^ rats presented more severe obesity than male rats and were approximately160% heavier at 8 months of age (Fig. 2C, n = 15, *P* = 0.02). The increased body weight of *Lepr*^−/−^ rats was associated with significantly elevated daily food consumption in both genders (Fig. 2D,E).

### Leptin receptor knockoutinduced hyperglycemia, glucose intolerance andhyperinsulinemia

**Figure 3:**
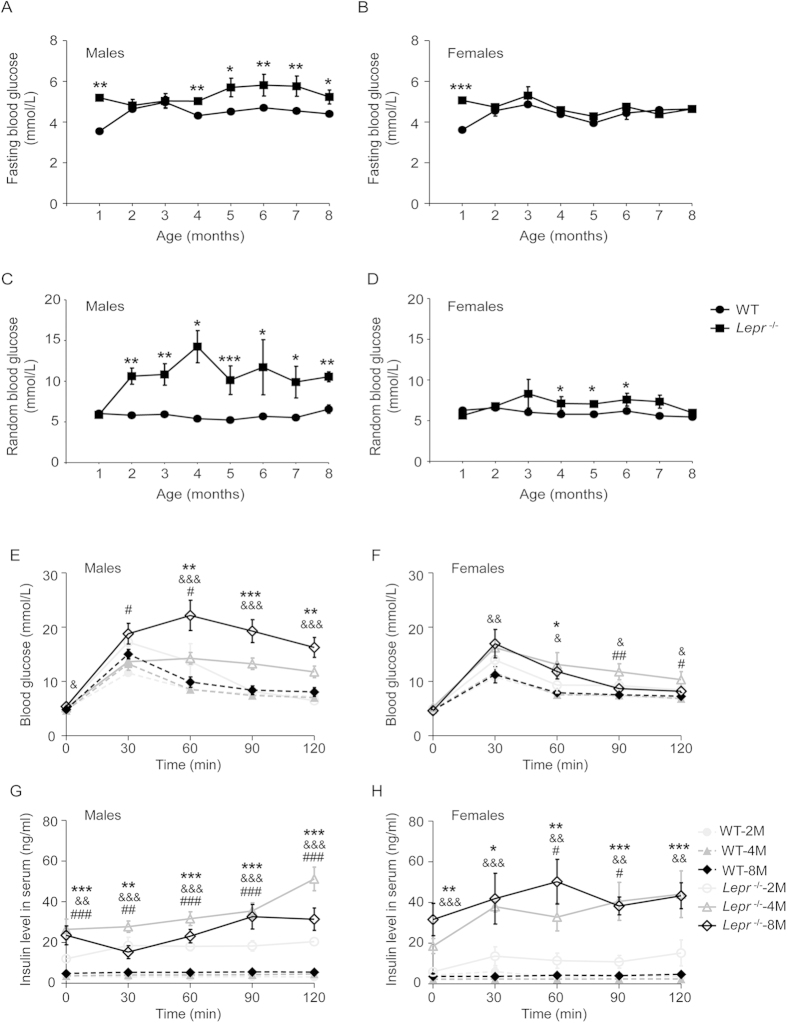
Blood glucose, glucose intolerance and serum insulin test of Leptin receptor knockout rat. (**A**–**D**) Fasting and random blood glucose were measured over 8 month for male WT littermate (n = 19), male *Lepr*^−/−^ rat (n = 12), female WT littermate (n = 13) and female *Lepr*^−/−^ rat (n = 15). (**E**,**F**) 2, 4 and 8months of age WT littermate and *Lepr*^−/−^ rat (n = 8 for each group) in both genders were administrated with D-glucose, and serum glucose levels were determined at 0, 30, 60, 90 and 120 min after administration. (**G**,**H**) Serum insulin levels of WT littermate and *Lepr*^−/−^ rat (n = 4 for each group) in both genders were measured in glucose tolerance tests meanwhile. *P < 0.05, **P < 0.01, ***P < 0.001 *versus* WT littermates in Figure 3A-3D; # refers to differences between WT-2M and *Lepr*^−/−^-2M; & refers to differences between WT-4M and *Lepr*^−/−^-4M and * refers to differences between WT-8M and *Lepr*^−/−^-8M in Fig. 3E–H; #, & and *P < 0.05, ##, && and **P < 0.01, ###, &&& and ***P < 0.001.

Fasting and random glucose levels were measured from 1 to 8 months of age in both genders. The male *Lepr*^−/−^ rats emerged with higher fasting glucose level at 4 months of age and this hyperglycemia continued to 8 months of age (Fig. 3A). Significantly higher random glucose levels occurred as early as 2 months of age in male *Lepr*^−/−^ rats and reached peak glucose at 4 months of age, which increased 1.64-fold compared with those of their WT littermates (Fig. 3C, n = 12, *P* = 0.02). Random hyperglycemia also continued to 8 months of age. However, persistent hyperglycemia was not observed in the female *Lepr*^−/−^ rats (Fig. 3B,D).

According to the above-described glucose levels, we chose 2-, 4- and 8-month old rats on which to performa glucose tolerance test. After the glucose loading, the *Lepr*^−/−^ rats showed rapid and remarkable elevation of serum glucose levels, whereas serum glucose elevation was relatively slow and brief in their WT littermates. Glucose intolerance appeared in male*Lepr*^−/−^ rats at 2 months of age and deteriorated with aging. In particular, at 8 months of age the peak glucose level reached 22.2 mmol/L and the glucose level was sustained at 16.3 mmol/L at 120 min after glucose loading (Fig. 3E, n = 8, *P* = 0.002). In female *Lepr*^−/−^ rats, glucose intolerance only was observed at 4 months of age (Fig. 3F).

To further evaluate glucose homeostasis in *Lepr*^−/−^ rats, serum insulin level were measured in a glucose tolerance test. The *Lepr*^−/−^ rats showed significant hyperinsulinemia at baseline and presented a dramatic increase in serum insulin levels at 2, 4 and 8 months of age in both genders after the glucose loading (Fig. 3G,H).

### Leptin receptor knockout induced dyslipidemia

Diabetes-associated lipid metabolism parameters were measured at 2, 4 and 8 months of age in both genders. The *Lepr*^−/−^ rats showed similar lipid metabolism characteristics in both genders, so we merged the parameters of males and females (Table 1). Circulating triglycerides, total cholesterol and high density lipoprotein were all significantly increased in *Lepr*^−/−^ rats compared with those of their WT littermates at 2, 4 and 8 months of age. A diabetic condition only altered the low density lipoprotein in 8-month old *Lepr*^−/−^ rats significantly. Dyslipidemia appeared in *Lepr*^−/−^ rats at 2 months of age and deteriorated with aging.

### Leptin receptor knockout induced pathological changes of pancreas, liver, adipose tissue and kidney

**Figure 4:**
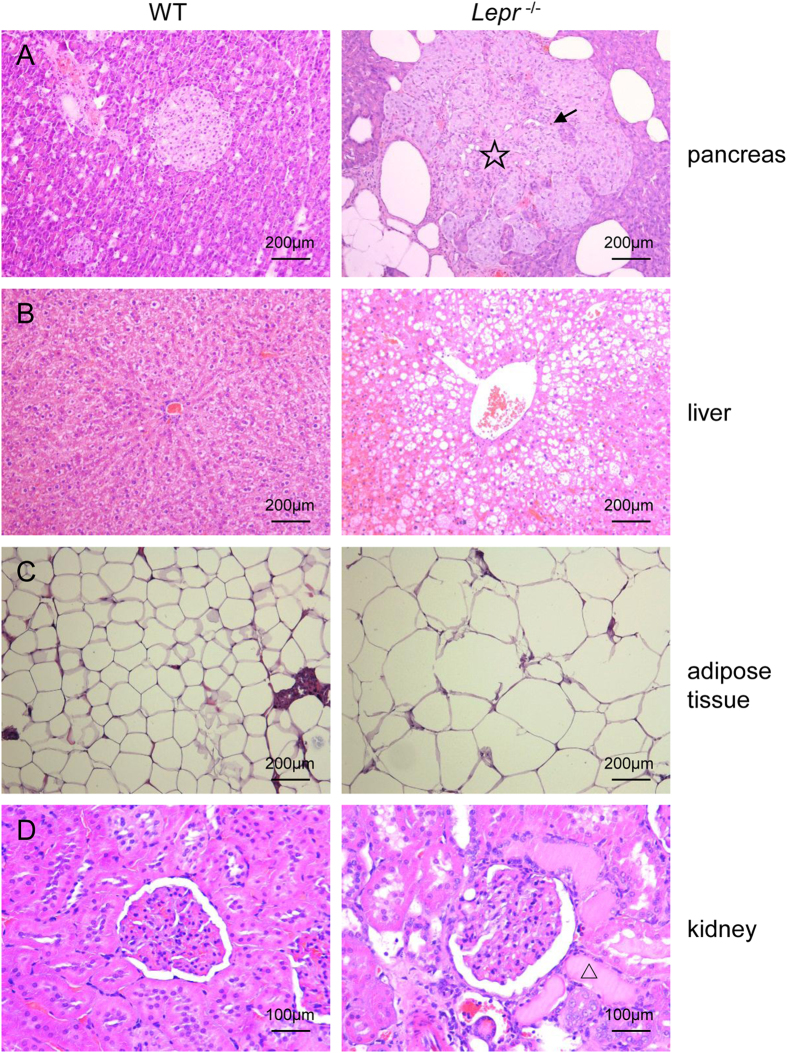
Pathological changes of pancreas, liver, adipose tissue and kidney in Leptin receptor knockout rat. (**A–C**) Haematoxylin and eosin (H&E) staining of the pancreas, liver and adipose tissue in WT littermates and the*Lepr*^−/−^ rats (magnification × 100); (**D**) H&E staining of the kidney in WT littermates and the *Lepr*^−/−^ rats (magnification × 200). ☆presents pancreas fibrosis, black arrows presents pancreas hemorrhage, △presents renal tubular damage.

The *Lepr*^−/−^ ratsand their WT littermates were chosen for observation of the pathological changes induced by *Lepr* knockout at 8 months of age. Compared with their WT littermates, the pancreatic islets of the *Lepr*^−/−^ rats exhibited obviously severe vacuolation, hypertrophy, fibrosis and hemorrhage, and had irregular boundaries (Fig. 4A). Infiltration of inflammatory cells was also observed in the islet (see Supplementary Fig. S3D). Severe hepatic steatosis, characterized by the accumulation of higher levels of lipid in the hepatic intracellular vesicles, was evident in the *Lepr*^−/−^ rats (Fig. 4B). The adipocytes of the *Lepr*^−/−^ rats were obviously larger (Fig. 4C). The kidney tissues of the *Lepr*^−/−^ rats exhibited expansion of glomerular matrix, segmental glomerulosclerosis and tubular damage such as tubular expansion and regeneration (Fig. 4D).

### Leptin receptor knockout induced the decrease of bone volume and bone mineral density

**Figure 5:**
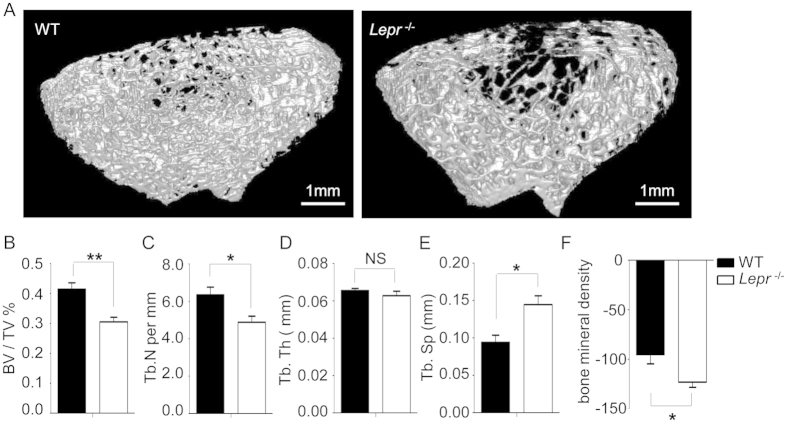
Bone volume and equivalent density analysis of Leptin receptor knockout rat. (**A**) μCT images of the trabecular bone compartment in the distal femur of the WT littermate and *Lepr*^−/−^ rats at 8 months of age (Scale bar: 1 mm). (**B**–**F**) Bone volume/total (tissue) volume (BV/TV), trabecular number (Tb.N), trabecular thickness (Tb.Th), trabecular separation (Tb.Sp) and bone mineral density (BMD) were measured in the WT littermates and *Lepr*^−/−^ rats at 8 months of age (n = 4 for each group, *P < 0.05, **P <* *0.01 *versus* WT littermates; NS: no significant difference).

WT littermates and *Lepr*^−/−^rats were euthanized (n = 4 for each group) at 8 months of age. Their femurs were dissected and the distal femur were analyzed by μCT. The trabecular bone of the distal femur was significantly decreased in the *Lepr*^−/−^ rats compared with that of WT littermates at 8 months of age (Fig. 5A). The 3-D analysis indicated that the bone volume/total (tissue) volume (BV/TV) of the *Lepr*^−/−^ rats was decreased by 26.40% (Fig. 5B, *P* = 0.006), and the trabecular number (Tb.N) was decreased by 23.08% (Fig. 5C, *P* = 0.03), whereas trabecular separation (Tb.Sp) was increased by 53.73% (Fig. 5E, *P* = 0.02). The bone mineral density (BMD) of the distal femur was significantly decreased by 29.35% in the *Lepr*^−/−^ rats compared with their WT littermates (Fig. 5F, *P* = 0.02).

## Discussion

In the past few decades, obesity and obesity-related disease, such as type 2 diabetes, have been shown to be directly related to increased mortality and reduced life expectancy^20^. Human type 2 diabetes is a complex heterogeneous disease; it is clinically characterized by obesity, overt hyperglycemia, dyslipidemia and glucose intolerance^21^. Moreover, long-term poor glycemic control in diabetic patients leads to the development of microvascular and macrovascular complications^22^,^23^. Patients with type 2 diabetes also present with a higher potential for falls and risk for fracture than nondiabetic individuals^24^,^25^.

Various animal models of type 2 diabetes have been established to study human type 2 diabetes. However,these models do not develop the full phenotype of type 2 diabetes. Thus, another animal model, in particular a rat model, due to its physiology advantages compared with mice, should be helpful for *Lepr*’s clinical applications.

Here, we created the*Lepr* knockout rats (named as *Lepr*^tm1Ilas^ in our laboratory Rat Resource website: http://123.1.153.158/portal/root/website_yky) using CRISPR/Cas9 technology.The phenotypes of the*db*/*db* mice, the Zucker rats and the*Lepr*^−/−^ rats was summarized in Table 2. Our two gRNAs targeting the fourth exon of the *Lepr* gene, induced a 298-bp deletion and a 4-bp insertion and resulted TGA termination codon prematurely, which led to the absence of LEPR in the *Lepr*^−/−^ rats. However, the Zucker rat model had a spontaneous missense mutation in the *Lepr* gene, which does not affect the expression level of LEPR^26^. The differences in LEPR expression resulted in disparate phenotypes between our *Lepr*^−/−^ rats and the Zucker rats. The *Lepr*^−/−^ rats are obese and develop mild random hyperglycemia, hyperinsulinemia and dyslipidemia as early as 8 weeks of age. Whereas, the Zucker rats do not present with typical high blood glucose levels^10^, and they develop glucose intolerance and insulin resistance within 12 weeks of age in a mixed genetic background (a cross between Merck 3M and Sherman rats)^27^, and demonstrate a delayed onset of glucose intolerance to 21–23 weeks when bred into the SD background^28^. Consistent with the Zucker rats, our *Lepr*^−/−^ rats demonstrated a significant decrease in BV/TV compared with their WT littermates^29^. Leptin has been found to restrain corticotropic releasing hormone and to stimulate gonadotropin releasing hormone release from the hypothalamus^30^. Leptin-deficiency or *Lepr*-deficiency obese animals are both hypercortisolic and hypogonadal^3^. Increased corticosteroid production and decreased estrogen presence favor an increase in osteoclast number and subsequent increase in bone resorption, which may predominate in the *Lepr*^−/−^ rats to increase the bone loss.

Another commonly used animal model of type 2 diabetes is *db*/*db* mice, which are characterized by hyperphagia, morbid obesity and extreme insulin resistance^5^,^31^. The hyperglycemia of *db*/*db* mice depends on the background strain. For example, *db*/*db*mice present with transient hyperglycemia in a C57BL/6J genetic background; although *db*/*db* mice in C57BL/KSJ genetic background exhibit uncontrollable hyperglycemia, they could only survive to 10 months of age^32^. Our *Lepr*^−/−^ rat presents with mild hyperglycemia as early as 1 month of age and this higher levels continued to 8 months of age. The chronic hyperglycemia of the *Lepr*^−/−^ rat demonstrates the advantage of long-term observation on the development of diabetes and diabetes-related complications. The *db*/*db* mice and the *Lepr*^−/−^ rat can possible complement each other in research on the development of diabetes.

In conclusion, our initial characterization shows that knockout of the *Lepr* gene in SD rats leads to severe obesity, hyperphagia, glucose intolerance, hyperinsulinemia, dyslipidemia, decreased bone mineral density and partial diabetes complications. Our model compensates for some deficiencies of the existing rodent models, especially with respect to chronic hyperglycemia, and it is proven to be a usefulanimal model for obesity and diabetes research.

## Methods

The use of animals and all experimental protocol were approved by the Animal Care and Use Committees of  The Institute of Laboratory Animal Science of Peking Union Medical College (ILAS-GC-2010-044), including the establishment of the *Lepr*knockout rats, fasting and random glucose test, glucose tolerance test, serum insulin level test, serum biochemistry test, histological analysis and microcomputedtomography analysis. And all the methods were carried out in accordance with the approved guidelines mentioned above.

### Animals

The *Lepr* knockout rats were generated by CRISPR/Cas9 as described previously^33^. In brief, we designed two pairs ofsynthesized oligonucleotides for gRNA targeting on the exon 4 of *Lepr*, TAGGCAAATCATCTATAACTTC and AAACGAAGTTATAGATGATTTG; TAGGCTGAAAGCTGTCTTTCAG and AAACCTGAAAGACAGCTTTCAG, which were annealed and cloned into the pUC57-gRNA expression vector (Plasmid #51132, Addgene, Cambridge, MA, USA, obtained from Professor Xingxu Huang). The gRNA expression plasmids were linearized with *Dra* I and used as templates for *in vitro* transcription using the MEGAshortscript Kit (Ambion, AM1354). The Cas9 expression plasmid (Plasmid #44758, Addgene, Cambridge, MA, USA, obtained from Professor Xingxu Huang) was linearized with *Age* I and used as the template for *in vitro* transcription using the T7 Ultra Kit (Ambion, AM1345). Transcribed Cas9 mRNA and gRNA were both purified by using the MEGAclear Kit (Ambion, AM1908), and then a mixture of transcribed Cas9 mRNA and gRNA was microinjected into Sprague Dawley (SD) rat (purchased from Beijing Vital River Laboratories Animal Center which were introduced from Charles River) zygotes to generate the *Lepr*^−/−^ rat. Microinjections were performed in the cytoplasm of zygotes using a Nikon Microinjection system under standard conditions. The rat was genotyped by PCR with the primers, 5′ CTTGTGTCCAGAGCCTTCCTATAAC and 5′ ATTCCCCATGTTGTCTAGTAGTGATC. For genotyping, a 662-bp fragment of WT and a 368-bp fragment of the *Lepr* knockout gene were amplified with 30 PCR cycles consisting of94 °C for 30 s, 60 °C for 30 s and 72 °C for 45 s.

All rats used in this study were maintained on a SD genetic background and were bred in an AAALAC-accredited facility. Rats were housed in a room kept at 23 ± 2 °C with a 12:12 h light/dark cycle and were provided with standard food and water libitum.The use of animals was approved by the Animal Care and Use Committees of The Institute of Laboratory Animal Science of Peking Union Medical College (ILAS-GC-2010-044).

### Protein Extraction and Western Blot Analysis

The rats were euthanized and total protein lysates from the rat liver tissues were prepared as previously described^34^. After SDS-PAGE and transfer of the bands to nitrocellulose (Millipore), the membranes were incubated overnight with antibodies against LEPR (Santa Cruz, sc-8325). After incubation with the appropriate secondary antibody for 1h at room temperature, antibody binding was detected with an HRP-conjugated immunoglobulin G (Santa Cruz) using a chemiluminescence detection system (Santa Cruz). For quantitative analysis, the LEPR level was normalized to β-actin.

### Body Weight and Food Consumption

WT littermates and *Lepr*^−/−^ rats of both genders were weighed every month from 1 to 8 months of age. WT littermates and *Lepr*^−/−^ rats were provided with standard food and water libitum. Food was weighed, and the average daily intake was calculated from 1 to 8 months of age.

### Fasting and Random Blood Glucose

WT littermates and *Lepr*^−/−^ rats of both genders were fasted overnight (14 h) but given water libitum. Blood was collected by tail vein puncture and blood glucose was analyzed by a One Touch Ultra glucometer (YZB/USA 6891). Random blood glucose measurement was performed at 9:00 a.m. over 8 month in both genders.

### Glucose Tolerance Test and Serum Insulin Level

WT littermates and *Lepr*^−/−^ rats of both genders were fasted overnight (14 h) but given water libitum. On the day of the test, the rats were weighed, and blood was collected by tail vein puncture. Blood glucose was analyzed by a One Touch Ultra glucometer (YZB/USA 6891). After a baseline glucose concentration was obtained, the rats were injected intraperitoneally with D-glucose at 1 g/kg body weight. Blood glucose levels were sampled from the tail at 30, 60, 90 and 120 min after injection. Meanwhile, 100 μl of blood was collected for serum insulin level test using rat/mouse insulin ELISA kits (Millipore).

### Serum Biochemistry

WT littermates and *Lepr*^−/−^ rats of both genders were fasted overnight (14 h) but given water libitum. Blood was collected by tail vein puncture. Whole blood was centrifuged at 3000 g for 10 min at 4 °C to obtain the serum and prepared for serum total cholesterol (CHO), triglycerides (TG), high density lipoprotein (HDL) and low density lipoprotein (LDL) detection using a HITACHI 7100 Automatic Analyzer.

### Histological Analysis

For light microscopy, the rats were euthanized, and the pancreas, liver, kidney and abdominal adipose tissue were fixed in 4% formaldehyde and mounted in paraffin blocks. The sections were stained with haematoxylin and eosin (H&E) and analyzed using the Aperio Image Scope v8.2.5 software. The sections were analyzed by an observer blinded to the rat genotypes.

### Microcomputed Tomography (μCT) Analysis

WT littermates and *Lepr*^−/−^ rats 8 months of age were euthanizedand their femurs were dissected. Measurements of trabecular architecture were performed on the distal femur cleared of all soft tissue using Siemens INVEON LG CT. After an initial scout scan, a total of 100 slices with an increment of 10 μm were obtained on each bone sample, starting 1.0 mm below the growth plate. The area for analysis was outlined within the trabecular compartment, excluding the cortical and subcortical bone. Every 5 sections were outlined, and the intermediate sections wereinterpolated with the contouring algorithm to create a volume of interest. Segmentation values used for analysis were determined using Inveon Research Workplace. A three-dimensional (3-D) analysis was performed to determine BV/TV, trabecular number (Tb.N), trabecular thickness (Tb.Th) and trabecular separation (Tb.Sp). A two-dimensional (2-D) analysis was performed to determine bone mineral density (BMD). The mean cortical thickness (Ct.Th) was determined by distance measurements at 4 different points on the cortical slice.

### Statistical Analysis

The data were analyzed by Two Independent-Samples non-parametric test. The data were expressed as the means ± SEMs from individual experiments. The differences were considered significant at *P* < 0.05.

## Additional Information

**How to cite this article**: Bao, D. *et al.* Preliminary Characterization of a Leptin Receptor Knockout Rat Created by CRISPR/Cas9 System. *Sci. Rep.* **5**, 15942; doi: 10.1038/srep15942 (2015).

## Supplementary Material

Supplementary Information

## Figures and Tables

**Table 1:** Fasting serum biochemistry at 2, 4 and 8 months of age.

Group	2M		4M		8M	
	WT	*Lepr*^−/−^	WT	*Lepr*^−/−^	WT	*Lepr*^−/−^
Number	11	6	8	9	7	7
CHO (mM)	1.56 ± 0.73	2.46 ± 0.56 *	1.69 ± 0.76	5.89 ± 1.04 ***	1.94 ± 0.76	8.15 ± 2.70 ***
TG (mM)	0.81 ± 0.39	2.84 ± 0.90 ***	1.16 ± 0.55	5.00 ± 2.08 ***	1.20 ± 0.78	6.26 ± 3.08 **
HDL (mM)	1.11 ± 0.55	1.78 ± 0.63 *	0.91 ± 0.49	3.73 ± 0.95 ***	1.44 ± 0.63	5.89 ± 2.28 ***
LDL (mM)	0.21 ± 0.10	0.32 ± 0.22	0.23 ± 0.12	0.29 ± 0.07	0.21 ± 0.05	0.76 ± 0.27 ***

CHO, total cholesterol; TG, triglycerides; HDL, high density lipoprotein; LDL, low density lipoprotein.

*P < 0.05, **P < 0.01, *** P < 0.001 *versus* WT littermate.

**Table 2:** Comparison of the phenotypes among *db*/*db* mice, the Zucker rats and our *Lepr* ^−/−^ rats.

	*db*/*db* mice	Zucker rats	*Lepr*^−/−^rats
*Lepr* disruption	Mutation at exon 19 g→t^35^,^36^ resulted in the deletion of the intracellular domain of *Lepr*.	Mutation at A^880^→C resulted in the replacement of Gln^269^→Pro^37^, which does not affect the expression level of LEPR^26^.	*Lepr* exon 4, 298-bp deletion and 4-bp insertion, generated stop codon, and leads the absence of LEPR expression.
Obesity	Appears at 1 month of age	Appears at 1 month of age	Appears at 1 month of age
Hyperphagia	Yes	Yes	Yes
Hyperglycemia	C57BL/6J background present with transient hyperglycemia; C57BL/KSJ background exhibit hyperglycemia, only survive to 10 months of age^32^.	Normal blood glucose levels^10^.	Mild hyperglycemia during 8 months of age.
Glucose intolerance	Yes	Yes, delayed onset of glucose intolerance^28^.	Yes, appears as early as 2 months of age.
Diabetic complication	Renal lesion;^38^ increased bone formation^3^.	Renal lesion; decreased bone volume^29^.	Pancreas, liver and renal lesion; decreased bone volume.
