# Supplementary material for: Preliminary Characterization of a Leptin Receptor Knockout Rat Created by CRISPR/Cas9 System
Source: Sci Rep. 2015 Nov 5;5:15942. doi: 10.1038/srep15942 (PMC4633582; doi:10.1038/srep15942)
Supplement: Supplementary Information [file srep15942-s1.pdf]

Preliminary Characterization of a Leptin Receptor Knockout Rat Created by CRISPR/Cas9 System

Dan Bao [a], Yuanwu Ma [a], Xu Zhang [a], Feifei Guan [a], Wei Chen [a], Kai Gao [a], Chuan Qin [b, *], Lianfeng Zhang [a, *]

[a] Key Laboratory of Human Disease Comparative Medicine, Ministry of Health, Institute of Laboratory Animal Science, Chinese Academy of Medical Sciences & Comparative Medical Center, Peking Union Medical College, China.

[b] Key Laboratory of Human Disease Animal Model, State Administration of Traditional Chinese Medicine, Institute of Laboratory Animal Science, Chinese Academy of Medical Sciences & Comparative Medical Center, Peking Union Medical College, China.

[*] Corresponding author:

Lianfeng Zhang, Ph.D.

Chuan Qin, Ph.D.

Building 5, Panjiayuan Nanli, Chaoyang District, Beijing 100021 China.

E-mail: Zhanglf@cnilas.org; Phone: 86-010-87778442; Fax: 86-010-67776394.

Supplementary Figures

Figure S1

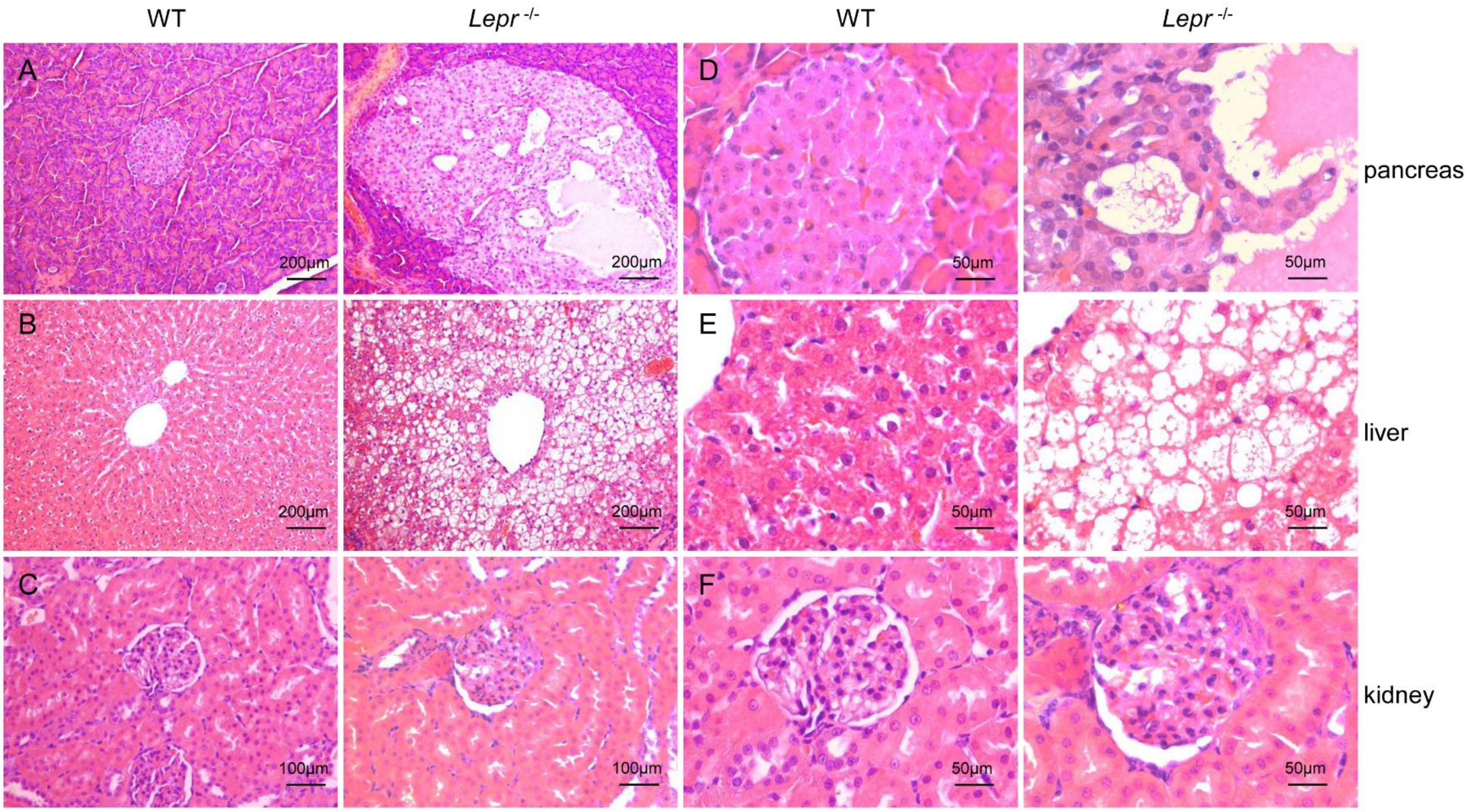

Supplementary Figure S1. Pathological changes of pancreas, liver and kidney in leptin receptor knockout rat at 2 months of age. (A-B) Haematoxylin and eosin (H&E) staining of the pancreas and liver in WT littermates and the *Lepr*$^{-/-}$ rats (magnification × 100); (C) H&E staining of the kidney in WT littermates and the *Lepr*$^{-/-}$ rats (magnification × 200); (D-F) H&E staining of the pancreas, liver and kidney in WT littermates and the *Lepr*$^{-/-}$ rats (magnification × 400).

Figure S2

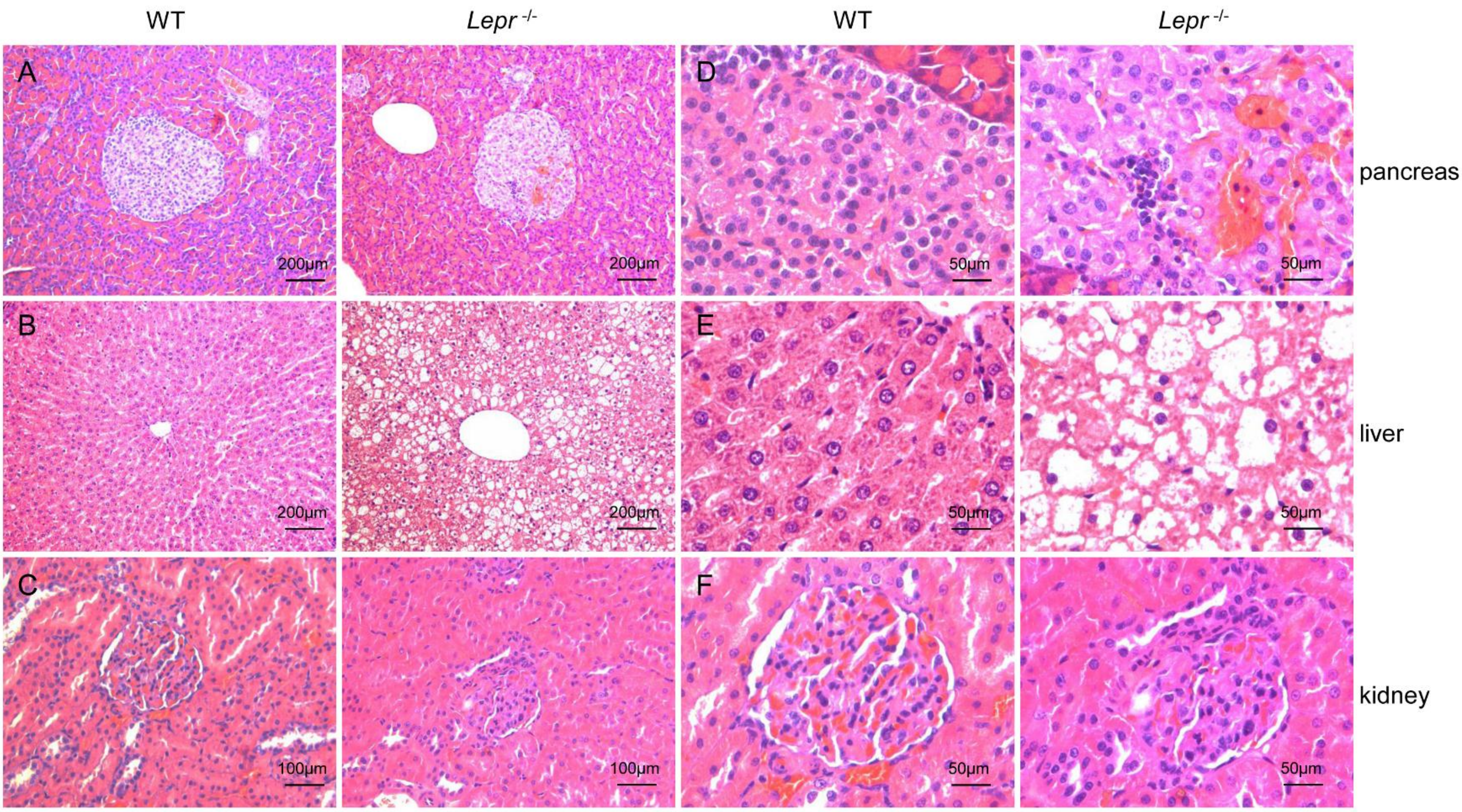

Supplementary Figure S2. Pathological changes of pancreas, liver and kidney in leptin receptor knockout rat at 4 months of age. (A-B) Haematoxylin and eosin (H&E) staining of the pancreas and liver in WT littermates and the *Lepr*$^{-/-}$ rats (magnification × 100); (C) H&E staining of the kidney in WT littermates and the *Lepr*$^{-/-}$ rats (magnification × 200); (D-F) H&E staining of the pancreas, liver and kidney in WT littermates and the *Lepr*$^{-/-}$ rats (magnification × 400).

Figure S3

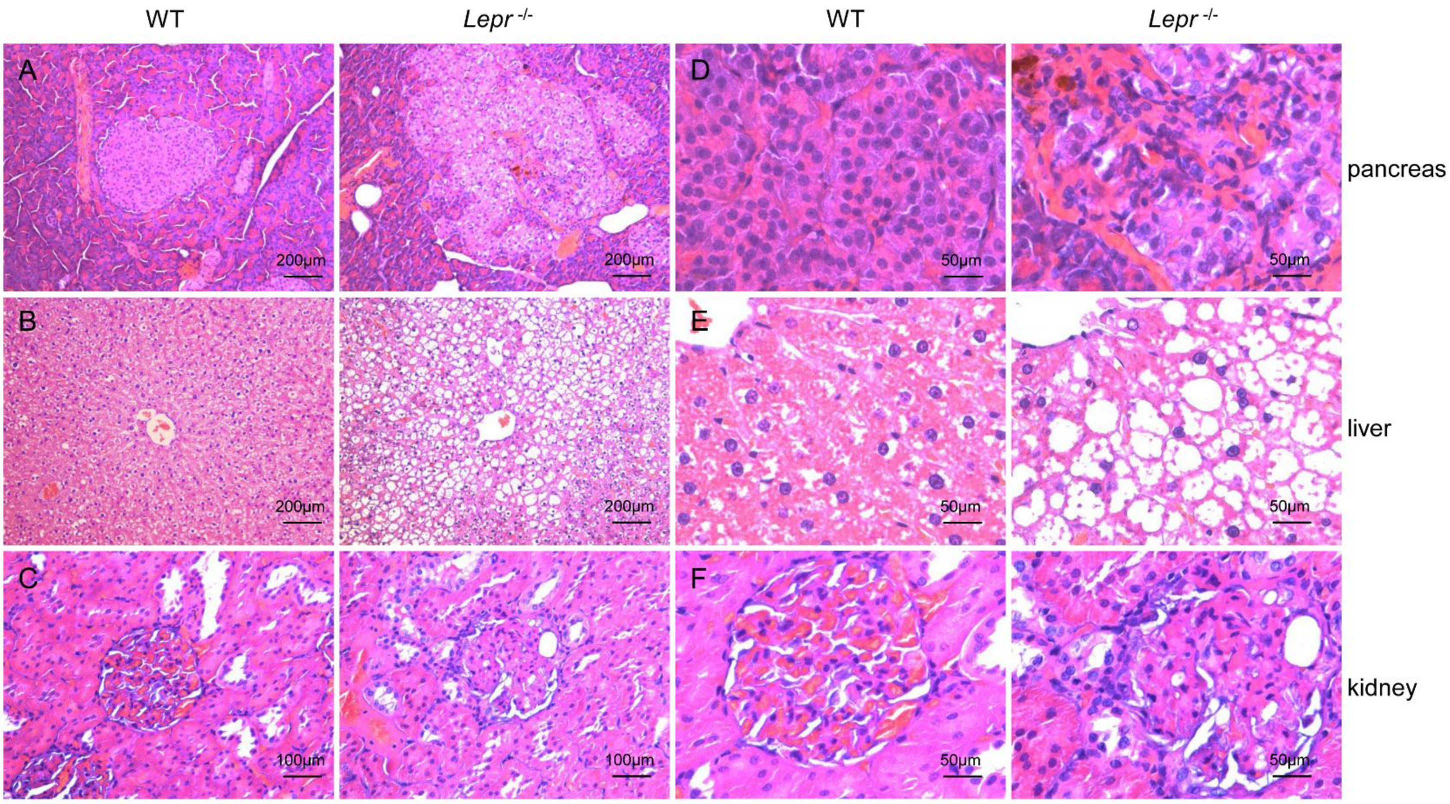

Supplementary Figure S3. Pathological changes of pancreas, liver and kidney in leptin receptor knockout rat at 8 months of age. (A-B) Haematoxylin and eosin (H&E) staining of the pancreas and liver in WT littermates and the *Lepr*$^{-/-}$ rats (magnification × 100); (C) H&E staining of the kidney in WT littermates and the *Lepr*$^{-/-}$ rats (magnification × 200); (D-F) H&E staining of the pancreas, liver and kidney in WT littermates and the *Lepr*$^{-/-}$ rats (magnification × 400).
